# Association between psychiatric disorders and iron deficiency anemia among children and adolescents: a nationwide population-based study

**DOI:** 10.1186/1471-244X-13-161

**Published:** 2013-06-04

**Authors:** Mu-Hong Chen, Tung-Ping Su, Ying-Sheue Chen, Ju-Wei Hsu, Kai-Lin Huang, Wen-Han Chang, Tzeng-Ji Chen, Ya-Mei Bai

**Affiliations:** 1 Department of Psychiatry, Taipei Veterans General Hospital, Taipei, Taiwan; 2 Department of Psychiatry, College of Medicine, National Yang-Ming University, Taipei, Taiwan; 3 Department of Family Medicine, Taipei Veterans General Hospital, Taipei, Taiwan; 4 Institute of Hospital and Health Care Administration, National Yang-Ming University, Taipei, Taiwan

**Keywords:** Iron deficiency anemia, Psychiatric disorders, Comorbidity

## Abstract

**Background:**

A great deal of evidence has shown that iron is an important component in cognitive, sensorimotor, and social-emotional development and functioning, because the development of central nervous system processes is highly dependent on iron-containing enzymes and proteins. Deficiency of iron in early life may increase the risk of psychiatric morbidity.

**Methods:**

Utilizing the National Health Insurance Database from 1996 to 2008, children and adolescents with a diagnosis of IDA were identified and compared with age and gender-matched controls (1:4) in an investigation of the increased risk of psychiatric disorders.

**Results:**

A total of 2957 patients with IDA, with an increased risk of unipolar depressive disorder (OR = 2.34, 95% CI = 1.58 ~ 3.46), bipolar disorder (OR = 5.78, 95% CI = 2.23 ~ 15.05), anxiety disorder (OR = 2.17, 95% CI = 1.49 ~ 3.16), autism spectrum disorder (OR = 3.08, 95% CI = 1.79 ~ 5.28), attention deficit hyperactivity disorder (OR = 1.67, 95% CI = 1.29 ~ 2.17), tic disorder (OR = 1.70, 95% CI = 1.03 ~ 2.78), developmental delay (OR = 2.45, 95% CI = 2.00 ~ 3.00), and mental retardation (OR = 2.70, 95% CI = 2.00 ~ 3.65), were identified. A gender effect was noted, in that only female patients with IDA had an increased OR of bipolar disorder (OR = 5.56, 95% CI = 1.98 ~ 15.70) and tic disorder (OR = 2.95, 95% CI = 1.27 ~ 6.86).

**Conclusion:**

Iron deficiency increased the risk of psychiatric disorders, including mood disorders, autism spectrum disorder, attention deficit hyperactivity disorder, and developmental disorders. Further study is required to clarify the mechanism in the association between IDA and psychiatric disorder.

## Background

According to the World Health Organization, iron deficiency (ID) is the most prevalent nutritional deficiency. A 30% prevalence of iron deficiency anemia (IDA), at a minimum, has been noted among children, adolescents, and women in non-industrialized countries, and ID is also the most prevalent nutritional deficiency in industrialized countries [1-4]. ID, defined by two or more abnormal measurements (serum ferritin, transferrin saturation, erythrocyte protoporphyrin), is insidious and uneasily detected by patients themselves and may not develop significant clinical symptoms [1-4]. IDA is characterized by a defect in hemoglobin synthesis owing to significant ID, resulting in the reduced capacity of the red blood cells to deliver oxygen to body cells and tissues, and many clinical symptoms, such as pale conjunctiva, shortness of breath, dizziness, and lethargy [1-4]. The main risk factors for IDA and ID include a low intake of iron, poor absorption of iron from diets, chronic loss of iron (i.e., ulcer, metrorrhagia), and some specific periods of life when iron requirements are especially high, such as growth and pregnancy [1-4].

Iron is an essential component of hemoglobin, myoglobin, and many enzymes in cellular metabolism and DNA replication and repair. It also plays a crucial role in the development of the central neurological system [5-8], autoimmune system [9-11], endocrine system [12-15], and cardiovascular system [16,17]. In the development of the brain, iron accounted for the myelination of white matter [18,19] and the development and functioning of the different neurotransmitter systems, including the dopamine, norepinephrine, and serotonin systems [20-22]. In vivo microdialysis studies using post-weaning iron-deficient rats and mice demonstrated that they exhibited deficits in intracellular dopamine concentrations and in the density of dopamine and dopamine transporter receptors, with variable amounts of loss by brain region [23-26]. Anderson et al. showed that decreased iron significantly reduced extracellular concentrations of norepinephrine in the caudate putamen, and altered levels of norepinephrine due to reduced iron levels may be the result of changes in the expression of norepinephrine transport and norepinephrine receptor proteins in the locus ceruleus and basal ganglia [27,28]. A reduction in serotonin transporter binding was noted in the nucleus accumbens and olfactory tubercle in iron-deficient rats [21], and the serotonin concentration in the brain was significantly correlated with the non-heme iron level [29].

In summary, IDA and ID were significantly associated with an alteration of monoamine neurotransmitters and the abnormal myelination of white matter, and is probably related to childhood/adolescence-onset psychiatric disorders. There is well documented evidence in the literature that IDA has a significant influence on cognitive development, intelligence, and developmental delay [30,31]. However, the association between IDA and other childhood/adolescence-onset psychiatric disorders is still limited. Some clinical studies supposed that brain ID involved in the pathophysiology of attention deficit hyperactivity disorder (ADHD) [32] and ferritin level was related to behavioral symptoms in ADHD patients [33]. But, Millichap et al. disclosed no significant difference in severity of ADHD symptoms between children with ADHD who had the lower serum ferritin levels (<20 ng/mL) and those who had the higher levels (>60 ng/mL) [34]. And a high prevalence, up to 30%, of IDA was observed in children with autism spectrum disorder (ASD), which was supposed to potentially compromise their communication and behavior [35,36]. As for schizophrenia, bipolar disorder (BD), unipolar depressive disorder, and anxiety disorder, the data are quite limited. This study, using a nationwide population-based insurance database with a case–control method and the largest sample size, attempted to clarify the association between IDA and various psychiatric disorders among children and adolescents with IDA. We hypothesized that children and adolescents with ADHD exhibited the higher risk of having a psychiatric disorder.

## Methods

### Data source

The National Health Insurance (NHI) program was implemented in Taiwan in 1995. Since 2001, Taiwan’s NHI has covered 96.9% of all 23,000,000 residents of Taiwan. The completeness and accuracy of the NHI claims database has been audited by the Department of Health and the Bureau of NHI. The database provides demographic and medical information on insured residents, including age, gender, prescription drugs, prescription date, and the prescription and diagnosis using the International Classification of Diseases, 9th Revision, Clinical Modification (ICD-9-CM). The NHI Research Database (NHIRD) has been used extensively in many epidemiologic studies in Taiwan [37-39]. Our study was approved by Institutional Review Board of Taipei Veterans General Hospital (2012-04-012BC).

### Inclusion criteria for IDA and psychiatric disorders

In this study, 1,000,000 subjects, approximately 4.3% of the population of Taiwan, were randomly selected from the NHIRD. The study comprised all children and adolescents (aged younger than 18) who were identified by the diagnostic code of “iron deficiency anemia” (ICD-9-CM: 280) between January 1, 1996 and December 31, 2008. Diagnosis was given by board-certificated pediatricians and physicians. Coexistent psychiatric disorders were investigated by specific diagnostic codes, and included schizophrenia (ICD-9-CM code: 295), BD (ICD-9-CM code: 296 except 296.2 and 296.3), unipolar depressive disorder (ICD-9-CM codes: 296.2, 296.3, 300.4, and 311 for major depressive disorder, dysthymic disorder, depressive disorder, not specified), obsessive-compulsive disorder (OCD) (ICD-9-CM code: 300.3), anxiety disorder (ICD-9-CM code: 300 except 300.3 and 300.4), ASD (ICD-9-CM code: 299), ADHD (ICD-9-CM code: 314), tic disorder (ICD-9-CM: 307.2), delayed development (ICD-9-CM code: 315), and mental retardation (ICD-9-CM code: 317 ~ 319). Finally, gastrointestinal ulcer (ICD-9-CM code: 531 ~ 534), metrorrhagia (ICD-9-CM code: 626.2 and 626.6), and premenopausal menorrhea (ICD-9-CM code: 627.0) were used as covariates. All psychiatric diagnoses were given by board-certified psychiatrists. The diagnoses of delayed development and mental retardation were given by board-certificated psychiatrists, rehabilitation specialists, and pediatricians.

### Control group

The age and gender-matched control group (4 for every patient in the study cohort) was randomly identified from subjects who had no major physical illnesses, including cancer (ICD-9-CM code: 140 ~ 239), hematological disease (ICD-9-CM code: 280 ~ 289), chronic liver disease (ICD-9-CM code: 570 ~ 573), chronic renal disease (ICD-9-CM code: 585, 586), chronic inflammatory disease (ICD-9-CM code: 710 for diffuse diseases of connective tissue, 714 for rheumatoid arthritis and other inflammatory polyarthropathies, 720 for ankylosing spondylitis and other inflammatory spondylopathies, 555 for Crohn’s disease, 556 for idiopathic proctocolitis), chronic infectious disease (ICD-9-CM code: 010 ~ 018 for tuberculosis, 042 ~ 044 for human immunodeficiency virus, 070 for viral hepatitis, 090 ~ 099 for syphilis, 390 ~ 392 for rheumatic fever, 730 for osteomyelitis), endocrine disease (ICD-9-CM code: 240 ~ 246 for thyroid disease, 249 ~ 250 for diabetes mellitus, 252 for parathyroid disease, 253 for pituitary disease, 255 for adrenal gland disease), and nutritional deficiency (ICD-9-CM code: 260 ~ 269).

### Statistical analysis

For between-group comparisons, the independent *t* test was used for continuous variables and Pearson's *X*^2^ test or Fisher's exact test was applied for nominal variables, where appropriate. Multiple logistic regressions were performed to calculate the OR with 95% confidence intervals (CI) after adjusting for ulcer, metrorrhagia, and premenopausal menorrhea (because ulcer and metrorrhagia were two important risks causing IDA of children and adolescents). A two-tailed *P*-value of less than 0.05 was considered statistically significant. All data processing and statistical analyses were performed with Statistical Package for Social Science (SPSS) version 17 software (SPSS Inc) and Statistical Analysis Software (SAS) version 9.1 (SAS Institute, Cary, NC).

## Results

### Demographic characteristics of the IDA patients and control group

A total of 2957 children and adolescents (1060 males and 1897 females) were identified as having IDA from the 1,000,000-person sample population between January 1, 1996 and December 31, 2008. The mean age was 10.59 ± 6.02 years and male patients were significantly younger than the females (7.46 ± 5.68 vs. 12.34 ± 5.46, p < 0.001). Male IDA patients exhibited a significantly higher prevalence of ASD (1.6% vs. 0.4%, p < 0.001), ADHD (6.0% vs. 1.1%, p < 0.001), tic disorder (1.3% vs. 0.5%, p = 0.027), delayed development (8.5% vs. 3.4%, p < 0.001), and mental retardation (4.4% vs. 1.5%, p < 0.001) than the females (Table 1). In comparing the difference between the IDA patients and the control group, the IDA patients had a significantly increased prevalence of psychiatric comorbidities, including unipolar depressive disorder (1.6% vs. 0.6%, p < 0.001), BD (0.4% vs. 0.1%, p < 0.001), anxiety disorder (1.5% vs. 0.7%, p < 0.001), ASD (0.8% vs. 0.3%, p < 0.001), ADHD (2.8% vs. 1.8%, p < 0.001), tic disorder (0.8% vs. 0.5%, p = 0.044), delayed development (5.2% vs. 2.4%, p < 0.001), and mental retardation (2.5% vs. 1.0%, p < 0.001) (Table 2).

**Table 1:** Characteristics of patients with iron deficiency anemia (IDA)

**Characteristics of IDA**	**Male**	**Female**	***p*-value**	**All**
No.	1060	1897		
Age (years)	7.46 ± 5.68	12.34 ± 5.46	<0.001	10.59 ± 6.02
Associated diseases, N (%)				
Schizophrenia	2 (0.2)	6 (0.3)	0.719	8 (0.3)
Unipolar depressive disorder	12 (1.1)	36 (1.9)	0.104	48 (1.6)
Bipolar disorder	2 (0.2)	11 (0.6)	0.154	13 (0.4)
OCD	1 (0.1)	3 (0.2)	>0.999	4 (0.1)
Anxiety disorder	21 (2.0)	24 (1.3)	0.158	45 (1.5)
ASD	17 (1.6)	7 (0.4)	0.001	24 (0.8)
ADHD	64 (6.0)	20 (1.1)	<0.001	84 (2.8)
Tic disorder	14 (1.3)	9 (0.5)	0.027	23 (0.8)
Delayed development	90 (8.5)	64 (3.4)	<0.001	154 (5.2)
Mental retardation	47 (4.4)	28 (1.5)	<0.001	75 (2.5)

*OCD* obsessive-compulsive disorder, *ASD* autistic spectrum disorder, *ADHD* attention-deficit hyperactivity disorder.

**Table 2:** Characteristics of patients with iron deficiency anemia (IDA) and control subjects

**Characteristics**	**IDA**	**Control**	***p*-value**
No.	2957	11828	
Age (years)	10.59 ± 6.02	10.59 ± 6.02	
Sex, N (%)			
Male	1060 (35.8)	4240 (35.8)	
Female	1897 (64.2)	7588 (64.2)	
Associated diseases, N (%)			
Schizophrenia	8 (0.3)	14 (0.1)	0.063
Unipolar depressive disorder	48 (1.6)	66 (0.6)	<0.001
Bipolar disorder	13 (0.4)	7 (0.1)	<0.001
OCD	4 (0.1)	8 (0.1)	0.274
Anxiety disorder	45 (1.5)	80 (0.7)	<0.001
ASD	24 (0.8)	32 (0.3)	<0.001
ADHD	84 (2.8)	207 (1.8)	<0.001
Tic disorder	23 (0.8)	56 (0.5)	0.044
Delayed development	154 (5.2)	278 (2.4)	<0.001
Mental retardation	75 (2.5)	114 (1.0)	<0.001

*IDA* iron deficiency anemia, *OCD* obsessive-compulsive disorder, *ASD* autistic spectrum disorder, *ADHD* attention-deficit and hyperactivity disorder.

### Odds ratio of psychiatric disorders

In examining the association between IDA and childhood/adolescence-onset psychiatric disorders, multiple logistic regression analysis was used to evaluate the OR of psychiatric comorbidity among those with IDA, after adjusting for age, gender, ulcer, metrorrhagia, and premenopausal menorrhea. Patients with IDA were prone to having a significantly higher chance of being associated with unipolar depressive disorder (OR = 2.34, 95% CI = 1.59 ~ 3.46), BD (OR = 5.80, 95% CI = 2.24 ~ 15.05), anxiety disorder (OR = 2.17, 95% CI = 1.49 ~ 3.16), ASD (OR = 3.08, 95% CI = 1.79 ~ 5.28), ADHD (OR = 1.67, 95% CI = 1.29 ~ 2.17), tic disorder (OR = 1.70, 95% CI = 1.03 ~ 2.78), delayed development (OR = 2.45, 95% CI = 2.00 ~ 3.00), and mental retardation (OR = 2.70, 95% CI = 2.00 ~ 3.65) (Table 3). We also investigated the gender effect on IDA and associated psychiatric comorbidities (Table 3). A significantly increased risk of unipolar depressive disorder (OR = 2.36, 95% CI = 1.10 ~ 5.10), anxiety disorder (OR = 1.73, 95% CI = 1.02 ~ 2.94), ASD (OR = 2.66, 95% CI = 1.41 ~ 5.00), ADHD (OR = 1.51, 95% CI = 1.12 ~ 2.04), delayed development (OR = 1.95, 95% CI = 1.50 ~ 2.53), and mental retardation (OR = 3.09, 95% CI = 2.08 ~ 4.61) were observed in male IDA patients in comparison with females, while a significantly higher risk of unipolar depressive disorder (OR = 2.37, 95% CI = 1.51 ~ 3.73), BD (OR = 5.56, 95% CI = 1.98 ~ 15.70), anxiety disorder (OR = 2.69, 95% CI = 1.57 ~ 4.62), ASD (OR = 4.07, 95% CI = 1.39 ~ 11.90), ADHD (OR = 2.00, 95% CI = 1.14 ~ 3.49), tic disorder (OR = 2.95, 95% CI = 1.27 ~ 6.86), delayed development (OR = 3.48, 95% CI = 2.48 ~ 4.88), and mental retardation (OR = 2.13, 95% CI = 1.34 ~ 3.40) were noted in female IDA patients compared with males (Table 3).

**Table 3:** Association between iron deficiency anemia (IDA) and psychiatric disorders

**OR (95% CI)**	**Male**	**Female**	**Children (Age <13)**	**Adolescent (Age≧13)**	**All**
Schizophrenia	2.20 (0.40, 12.02)	2.15 (0.76, 6.10)	4.35 (0.27, 69.56)	2.04 (0.79, 5.28)	2.11 (0.86, 5.15)
Unipolar depressive disorder	**2.36 (1.10, 5.10)**	**2.37 (1.51, 3.73)**	1.33 (0.58, 3.04)	**2.89 (1.84, 4.54)**	**2.34 (1.59, 3.46)**
Bipolar disorder	8.80 (0.80, 97.14)	**5.56 (1.98, 15.70)**	5.20 (0.83, 32.71)	**6.05 (1.97, 18.58)**	**5.80 (2.24, 15.05)**
OCD	0.78 (0.08, 7.48)	3.07 (0.67, 14.13)	--	3.01 (0.77, 11.77)	1.87 (0.55, 6.40)
Anxiety disorder	**1.73 (1.02, 2.94)**	**2.69 (1.57, 4.62)**	1.29 (0.74, 2.26)	**3.71 (2.16, 6.37)**	**2.17 (1.49, 3.16)**
ASD	**2.66 (1.41, 5.00)**	**4.07(1.39, 11.90)**	**3.00 (1.70, 5.27)**	2.55 (0.40, 16.24)	**3.08 (1.79, 5.28)**
ADHD	**1.51 (1.12, 2.04)**	**2.00 (1.14, 3.49)**	**1.50 (1.12, 1.99)**	**2.54 (1.31, 4.95)**	**1.67 (1.29, 2.17)**
Tic disorder	1.23 (0.66, 2.30)	**2.95 (1.27, 6.86)**	1.41 (0.81, 2.43)	**3.73 (1.12, 12.48)**	**1.70 (1.03, 2.78)**
Delay in development	**1.95 (1.50, 2.53)**	**3.48 (2.48, 4.88)**	**2.23 (1.79, 2.78)**	**3.88 (2.17, 6.95)**	**2.45 (2.00, 3.00)**
Mental retardation	**3.09 (2.08, 4.61)**	**2.13 (1.34, 3.40)**	**3.01 (2.09, 4.32)**	**2.08 (1.22, 3.57)**	**2.70 (2.00, 3.65)**

*OCD* obsessive-compulsive disorder, *ASD* autistic spectrum disorder, *ADHD* attention-deficit hyperactivity disorder.

Bold type denotes statistically significant odds ratio (OR).

### Odds ratio of psychiatric disorder stratified by age

We performed adjusted multiple logistic regression stratified by age to elucidate the association between IDA and various psychiatric comorbidities, focusing on children and adolescents with IDA, respectively (Table 3). Children were defined as those younger than 13 and adolescents as those aged 13 ~ 18. Children with IDA exhibited a significantly higher risk of ASD (OR = 3.00, 95% CI = 1.70 ~ 5.27), ADHD (OR = 1.50, 95% CI = 1.12 ~ 1.99), delayed development (OR = 2.23, 95% CI = 1.79 ~ 2.78), and mental retardation (OR = 3.01, 95% CI = 2.09 ~ 4.32) (Table 3). Adolescents with IDA were significantly associated with unipolar depressive disorder (OR = 2.89, 95% CI = 1.84 ~ 4.54), BD (OR = 6.05, 95% CI = 1.97 ~ 18.58), anxiety disorder (OR = 3.71, 95% CI = 2.16 ~ 6.37), ADHD (OR = 2.54, 95% CI = 1.31 ~ 4.95), tic disorder (OR = 3.73, 95% CI = 1.12 ~ 12.48), delayed development (OR = 3.88, 95% CI = 2.17 ~ 6.95), and mental retardation (OR = 2.08, 95% CI = 1.22 ~ 3.57) (Table 3).

## Discussion

The results of our clinical epidemiological study supported previous findings that IDA is significantly associated with increased risks of unipolar depressive disorder, BD, anxiety disorder, ASD, ADHD, delayed development, and mental retardation among children and adolescents.

### Mental retardation and delayed development

Our results showed a higher prevalence of mental retardation and delayed development among subjects with IDA, which was consistent with previous findings [31,40,41]. Beltrán-Navarro et al. used the Bayley Scales of Infant Development, preschool language scales and an environmental sound perception task to assess the effect of IDA on multifaceted development in infancy (at 6 and 14 to 18 months) and showed that infants with chronic IDA did show significantly lower scores on language, environmental sound perception, and motor measures, when compared with infants with a normal iron nutritional status [42]. Studying the long-term developmental outcome of infants with IDA, Lozoff et al. found lower scores on tests of mental (i.e., intelligence) and motor functioning at age of 5 and 12 among those with IDA in infancy [31,40]. Furthermore, IDA can impair cognitive performance at all stages of life. Halterman et al. assessed 5398 children and adolescents aged 6 to 16 and demonstrated that subjects with IDA had more than twice the risk of scoring below average in scholastic achievement than the children with a normal iron status [43].

### Attention deficit hyperactivity disorder (ADHD)

Previous studies on iron and the pathophysiology of ADHD have had inconsistent results. In a small cohort study comprising 52 ADHD children, Oner et al. found that lower ferritin levels were associated with higher hyperactivity scores in ADHD children, but did not have a significant influence on cognitive measures [33]. Using magnetic resonance imaging (MRI) to investigate the association of ADHD and brain iron levels in the putamen, pallidum, caudate, and thalamus, Cortese et al. demonstrated children with ADHD showed significantly lower brain iron levels in the right and left thalamus compared to healthy controls [32]. Other studies, however, did not find this association between ADHD and the ferritin level [34,44]. In our study, an increased risk of ADHD was noted among those with IDA, which was compatible with a recent meta-analysis result [45]. With regard to the current pathophysiology of ADHD as involving a dysfunction of the neurotransmitter systems, IDA significantly disturbed the development and functioning of norepinephrine and dopamine neurotransmitter systems.

### Autism spectrum disorder (ASD)

Previous studies have revealed that iron was associated with socio-cognitive and socio-emotional development and functioning [46-48]. A disorder in this area is one of the core symptoms of ASD. Assessing the prevalence of ID and IDA among 116 children between 3 and 16 years old with a diagnosis of ASD, Hergüner et al. revealed that 24% of patients with ASD had ID, and 15% had IDA [35]. Latif et al. analyzed the serum ferritin measurements of 96 children with ASD and suggested there was a high prevalence, up to 30 percent, of ID in children with ASD [36]. Our result showed an increased risk of ASD among children and adolescents with IDA, suggesting a possible reciprocal effect between ASD and IDA. For example, inappropriate eating habit in those subjects may be related to IDA and many children with ASD are picky eaters, which could contribute to IDA. Furthermore, diffusion-tensor MRI found impaired white matter integrity and a myelination developmental abnormality in ASD patients compared to normal subjects [49-51]. Iron was regarded as an essential component in myelination and oligodendrogenesis. Myelin synthesis was limited and altered by ID. Further study would be needed to clarify the comorbidity or causality between ASD and IDA.

### Unipolar depressive disorder

Numerous studies have reported that IDA was associated with unipolar depressive disorder, possibly through an alteration of the monoamine neurotransmitters by ID [52-54]. Lower serum ferritin concentrations were significantly correlated with depressive symptoms [53,54]. Using a longitudinal cohort study of 191 participants to evaluate the association between IDA in infancy and affective and developmental outcomes in adolescence, Lozoff et al. revealed that those with IDA in infancy exhibited more anxiety, depression, social problems, and attentional problems in later life [40]. Vahdat Shariatpanaahi et al. studied 192 female medical students and found the mean ferritin level of students with depression was significantly lower than that of healthy students [53]. Similar results were observed in a Japanese male population. In a study of 312 men, those with lower serum ferritin concentration levels had a higher prevalence of depressive symptoms [54]. Our result, showing an increased risk of unipolar depressive disorder among those with IDA, was compatible with that of previous studies, and reconfirmed the pathophysiological association of IDA with unipolar depressive disorder.

### Bipolar disorder (BD)

Emotional dysregulation has been deemed as one of the core neuropsychopathologies of BD [55-57]. It has been well documented that iron is associated with socio-emotional development and that ID may disturb the development of emotional regulation [40,46,47,58], although clinical studies on the association between IDA and BD are few. Our results showed a higher prevalence of BD among IDA subjects. Structural and functional changes related to emotional dysregulation such as in the dorsal and ventral prefrontal cortices and the prefrontal-subcortical and associated limbic circuitry have been considered as a neuropathology of BD [56,57]. ID has been related to perturbations in myelin formation, alterations of monoamine neurotransmitter systems, particularly in the striatum, and deficits in energy metabolism, particularly in the hippocampus and prefrontal cortex [59,60]. The effect of IDA on BD is still unclear and further study is needed to investigate the possible causality between IDA and BD. Furthermore, a significantly increased OR of BD was noted only among females with IDA after controlling gastrointestinal ulcer, metrorrhagia, and premenopausal menorrhea. Clarifying this gender effect between IDA and BD may be needed in future studies.

### Anxiety disorder

Clinical studies on the association between IDA and anxiety disorder are few. Lozoff et al. found that children with severe, chronic ID in infancy had a greater prevalence of anxiety, depression, and attention problems [40]. Some animal studies have shown an association between IDA and alterations in serotonin, norepinephrine, and gamma-aminobutyric acid (GABA) neurotransmission [21,61]. In assessing weanling rats with either an iron deficient diet or a control diet for 6 weeks, Beard et al. found reduced activity and increased anxiety-like behaviors among the iron deficient rats with significant decrements in brain iron content in the corpus striatum, prefrontal cortex, and midbrain [62]. Emotional dysregulation due to ID may be a possible mechanism explaining the association between anxiety and IDA [63-65]. In our study, both males and females with IDA had a significantly higher risk of anxiety disorder. However, establishing causality between IDA and anxiety disorder will require further study in the future.

Regarding emotional and cognitive problems, current neuroanatomical imaging studies have proven that brain development is a continuous process from infancy to late adolescence or early adulthood [66,67]. A deficiency of essential components will greatly impair the normal trajectory of brain development. Iron is regarded as one of the essential nutritional elements related to cognitive and socio-emotional development and functioning. A significantly higher risk of anxiety disorder, ASD, ADHD, delayed development, and mental retardation was noted among children (below age 13) with IDA. After entering adolescence (age 13 ~ 18), those with IDA had an increased risk of unipolar depressive disorder, BD, anxiety disorder, ADHD, delayed development, and mental retardation. These results may indicate that ID, whether in childhood or adolescence, does have a great impact on psychiatric comorbidities. Moreover, iron deletion in childhood has immediate and chronic effects on brain development, in that children with IDA exhibited an increased risk of delayed development and mental retardation with persistent sequelae of cognitive impairment and emotional problems during adolescence.

### Tic disorder

We found a significantly increased risk of tic disorder among females with IDA. The association between IDA and tic disorder is unclear and rarely reported in the literature. Cortese et al. mentioned a possible iron hypothesis in a possible spectrum disorder of ADHD, restless leg syndrome, and tic disorder, because these 3 disorders were sometimes comorbid and shared a pathogenesis similar to monoamine neurotransmitter dysfunction [68]. Exploring the associations of ferritin levels with regional brain volumes among patients with Tourette’s syndrome, Gorman et al. demonstrated ferritin and serum iron levels were significantly lower in the Tourette's syndrome subjects and that ferritin correlated positively with putamen volume [69]. Combining these results with ours may inspire further study to clarify the effect of iron on tic disorder or other movement disorders, especially in female populations.

Some limitations in our study should be mentioned here. First, the prevalence of psychiatric disorders was deemed underestimated because our results were derived from insurance registry-based data. Only those individuals who used the medical resource to seek psychiatric help were identified. However, patients included in our study were given a diagnosis by board-certified physicians and the diagnoses were more reliable than self-reported ones. Second, those subjects who were not diagnosed as having IDA ever but had iron deficiency problem cannot be detected in our study. The clinical study would be required to elucidate the possible association between psychiatric disorders and iron deficiency or subthreshold IDA. Third, ID may be due to inappropriate eating habits in some cases but the association among IDA, eating disorder, and episodic mood disorder with changed eating pattern is still unclear. In our study, we focused on the risks of psychiatric comorbidities in patients with IDA and the further study is required to analyze the effect of inappropriate eating pattern of specific psychiatric disorder on the risk of IDA. Fourth, we had no personal information that could contribute to an understanding of the risk of psychiatric disorders in patients, such as environmental factors (i.e. long-term life stress, traumatic experience), and family history of psychiatric disorder. Forth, the causality between IDA and psychiatric disorders cannot be proved in our study, even though our results did indicate a significant association between IDA and psychiatric disorders.

## Conclusions

In conclusion, patients with IDA did have a higher risk of psychiatric disorders, including unipolar depressive disorder, BD, anxiety disorder, ASD, ADHD, delayed development, and mental retardation. When encountering patients with IDA in clinical practice, prompt iron supplementation should be considered to prevent possible psychiatric sequelae, because ID does impair the development of emotional regulation and cognition. And vice versa, psychiatrists should check the iron level in those children and adolescents with psychiatric disorders. Finally, further well-designed cohort studies are needed to elucidate the causality or comorbid effect between IDA and psychiatric disorders.

## Competing interests

The authors declare that they have no competing interest.

## Authors’ contributions

MHC and YMB designed the study, wrote the protocol and manuscripts. YMB, TPS, YSC, JWH, and KLH contributed to the preparation and proof-reading of the manuscript. YMB, TJC, and WHC provided the advices on statistical analysis. All authors read and approved the final manuscript.

## Pre-publication history

The pre-publication history for this paper can be accessed here:

http://www.biomedcentral.com/1471-244X/13/161/prepub
